# Color-tunable mixed photoluminescence emission from Alq_3_ organic layer in metal-Alq_3_-metal surface plasmon structure

**DOI:** 10.1186/1556-276X-9-569

**Published:** 2014-10-13

**Authors:** Nai-Chuan Chen, Chung-Chi Liao, Cheng-Chang Chen, Wan-Ting Fan, Jin-Han Wu, Jung-Yu Li, Shih-Pu Chen, Bohr-Ran Huang, Li-Ling Lee

**Affiliations:** 1 Department of Electronic Engineering, Chang Gung University, Tao-Yuan 333, Taiwan; 2 Institute of Electro-Optical Engineering, Chang Gung University, Tao-Yuan 333, Taiwan; 3 Department of Electronic Engineering, National Taiwan University of Science and Technology, Taipei 106, Taiwan; 4 Graduate Institute of Electro-Optical Engineering, National Taiwan University of Science and Technology, Taipei 106, Taiwan; 5 Green Energy and Environment Research Laboratories, Industrial Technology Research Institute (ITRI), 195, Sec. 4, Chung-Hsin Road, Chutung 310, Taiwan

**Keywords:** Surface plasmon polariton, White light, Organic light-emitting, Photoluminescence

## Abstract

This work reports the color-tunable mixed photoluminescence (PL) emission from an Alq_3_ organic layer in an Au-Alq_3_-Au plasmonic structure through the combination of organic fluorescence emission and another form of emission that is enabled by the surface plasmons in the plasmonic structure. The emission wavelength of the latter depends on the Alq_3_ thickness and can be tuned within the Alq_3_ fluorescent spectra. Therefore, a two-color broadband, color-tunable mixed PL structure was obtained. Obvious changes in the Commission Internationale d’Eclairage (CIE) coordinates and the corresponding emission colors of Au-Alq_3_-Au samples clearly varied with the Alq_3_ thickness (90, 130, and 156 nm).

## Background

In recent years, organic light-emitting diodes (OLED) have been attracting considerable attention for various illumination applications because they exhibit excellent properties that traditional light sources do not, including high brightness, large size, transparency, and flexibility. OLED have been considered to be potential next generation of light sources [1-3]. To realize full color and white OLED, various color mixing structures, including multiple dopant emissive layers and multiple emissive layers, and down conversion in the optical microcavity, have been utilized with various degrees of success [4-10]. Some methods for generating white color emission from OLED have been developed, such as the method of partial energy transfer in which the OLED materials are doped with fluorescent/phosphorescent dyes [11-20]. The mixing of the EL from the host molecules with excimer/exciplex emissions has also been found to yield white emission [21-23]. Other methods involve stacking red-, green- and blue-emissive components on a charge generation layer (CGL) [24-29]. Recently, metal-dielectric-metal (MDM) surface plasmon (SP) structures, which are similar to planar optical microcavities, have attracted a great deal of attention because they have various potential applications in optoelectronic devices [30-34].

In an MDM structure with semi-infinitely thick metals, if the thickness of the dielectric is smaller than the SP penetration depth at the metal/dielectric interface, then the SPs on both interfaces of the dielectric layer can interact with each other and split into two hybridized SP modes: an odd SP with an anti-symmetric magnetic field distribution and an even SP with a symmetrical magnetic field distribution [30]. The dispersion curve of the odd SP can cross the light line in air and shift up (or down) in energy when the dielectric thickness is reduced (or increased), indicating that the odd SP is not only radiative when one of the two semi-infinite thick metal layers is reduced to a finite and semi-transparent layer but that its energy is also tunable by variation of the dielectric thickness. Therefore, if the dielectric layer in the MDM structure is replaced with an organic emitter layer with a thickness of the order of an optical wavelength, then owing to near-field optics and the Purcell effect, the organic excitons can preferentially recombine into the odd SP that is confined in this metal-organic emitter layer-metal structure, and the radiative odd SP subsequently couples out of the device to photons in the air. This property has been investigated experimentally [32,33].

However, the application of this light emission has so far received little attention. In this work, a color-tunable mixing method, using a single organic light-emitting layer that is embedded between two metal layers of finite thickness is proposed. When one of the two metals is of finite thickness and is semi-transparent, the organic fluorescence still occurs and serves as a constant source of light. Further light emission is produced through the odd SP, and the wavelength of this emission is tunable by variation of the thickness of the organic layer. Therefore, color-tunable mixed emission from an MDM structure with finite thickness metal layers can easily be realized.

## Methods

### Design and fabrication of sample

**Figure 1:**
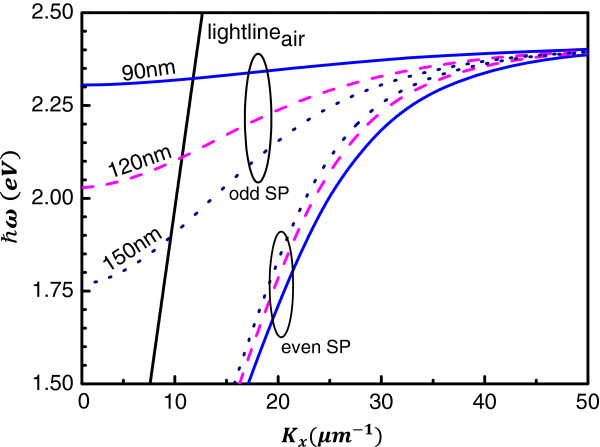
Dispersion curves of MDM structures with different dielectric thicknesses.

Because the dispersion curves of the coupled SPs in the MDM structure depend mainly on the thickness of the dielectric, calculated dispersion curves are initially obtained to determine the thickness of the dielectric that will produce the specific photon emission energy enabled by the odd SP. Figure 1 plots the calculated dispersion curves of the MDM structure with semi-infinite thick metal layers and dielectric thicknesses of 90, 120, and 150 nm. The refractive index of the dielectric layer is a constant 1.74, which is the refractive index of the organic material, tris(8-hydroxyquinoline) (Alq_3_), used in the experiment. The wavelength-dependent refractive index for the Au metal is taken from the literature [35]. As shown, the odd SP energies associated with the parallel component of the wave vector ***k***_***x***_ =0, which is the photon emission energy in the direction normal to the sample surface, are approximately 1.77 eV (700 nm), 2.03 eV (610 nm), and 2.30 eV (539 nm) for MDM structures with 150-, 120-, and 90-nm-thick dielectric layers, respectively.

**Figure 2:**
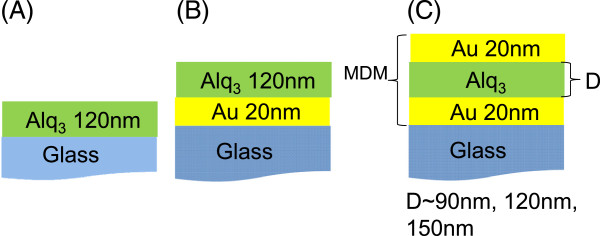
**Schematic structures of samples (A) Alq**_**3 **_**only, (B) single-metal, (C) MDM.**

Based on an analysis of the dispersion curve, three MDM samples, A, B, and C, with Alq_3_ thicknesses of 90, 120, and 150 nm, respectively, embedded between two 20-nm-thick Au layers were fabricated. Two reference samples, consisting of glass/120-nm-thick Alq_3_ and glass/20-nm-thick Au/120-nm-thick Alq_3_, were fabricated and are shown in Figure 2a,b, respectively. The organic material was subjected to temperature-gradient sublimation under a high vacuum before use. The organic and metal layers were deposited by vacuum vapor deposition in a vacuum chamber with a base pressure of <10^-6^ Torr. The deposition system permitted the fabrication of the complete device structure in a single vacuum pump-down without breaking the vacuum. The deposition rate of organic layers was kept at approximately 0.1 nm/s. The area of the MDM structure was 3 × 3 cm^2^, defined by a shadow mask. Figure 2c shows the MDM structure.

## Results and discussion

**Figure 3:**
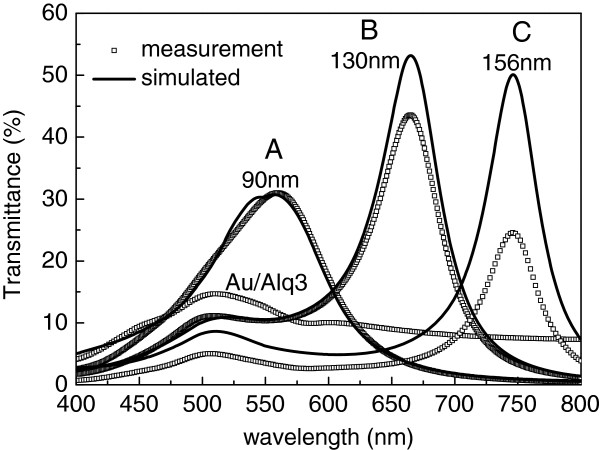
**Measured transmittance spectra from Au (20 nm)/Alq**_**3**_**/Au (20 nm) samples with different Alq**_**3 **_**layer thicknesses.** Results of theoretical simulations are shown.

Figure 3 shows the transmittance spectra of the three MDM samples with the different Alq_3_ thicknesses, measured using a Lambda 35 UV-vis spectrophotometer (PerkinElmer Inc., Waltham, MA, USA). Because both Au layers are sufficiently thin, externally incident light can be transmitted through the MDM structure by resonant transmission with the radiative optical modes that are present in the MDM structure. Therefore, the peaks that are induced by the odd SP can be observed in the transmittance spectra. To support the determination of the exact structure, the transmittance of a p-polarized plane wave in air, incident on 30-nm-thick Au/90, 130, and 156-nm-thick Alq_3_/30-nm-thick Au structures, was simulated using the software that was written by the authors and based on the solution for the boundary conditions of the electromagnetic waves, and the results thus obtained are also shown in Figure 3. The calculated and measured transmission peak positions match each other closely, and the transmitted wavelength depends sensitively on the thickness of Alq_3_ layer. From Figure 3, the wavelengths of the photons whose emission is enabled by the odd SP in the normal direction for samples A, B, and C are approximately 550, 650, and 750 nm, respectively.

**Figure 4:**
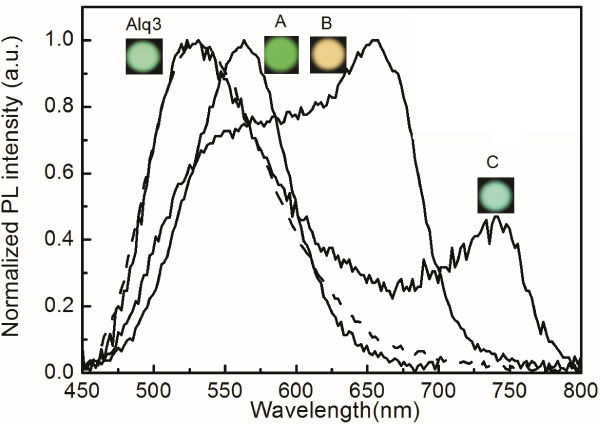
**PL spectra observed normal to the surfaces of samples A, B, and C and the reference sample.** Photographs of corresponding PL emission colors.

**Figure 5:**
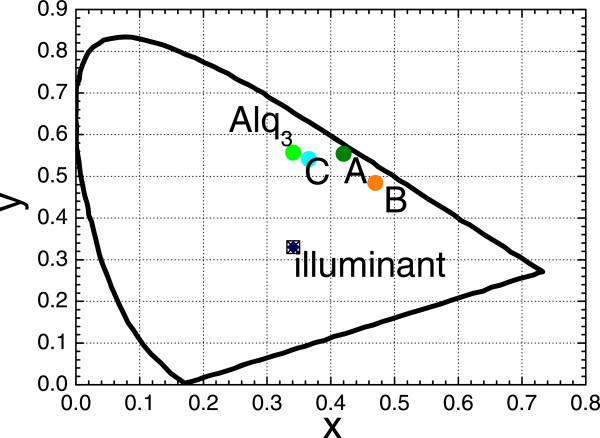
The CIE coordinates of emission samples A, B, C, and the reference sample.

Figure 4 shows the photoluminescence (PL) spectra in the normal direction from samples A, B, and C and from the reference sample with the glass/120-nm-thick Alq_3_ structure. As expected, the emission peaks around 550, 650, and 750 nm for samples A, B, and C closely match the peaks in the transmittance spectra in Figure 3. The PL intensity at the wavelength of 750 nm is smaller than that at 550 or 650 nm because the population of excitons with an energy that corresponds to 750 nm is smaller than that with an energy that corresponds to 550 or 650 nm. From the PL spectrum of sample C, the organic excitons recombine into the odd SP very well, even if the peak wavelength of approximately 750 nm is at the edge of the Alq_3_ fluorescent spectrum, while the emission peak at around 530 nm in the green region almost overlaps that in the PL spectrum from the reference sample, demonstrating that fluorescence from the Alq_3_ emissive layer is produced in MDM structures with metal layers of finite thickness.The Commission Internationale d’Eclairage (CIE) coordinates of the PL in the normal direction from samples A, B, and C and from the reference sample are (0.42, 0.55), (0.47, 0.48), (0.37, 0.54), and (0.34, 0.56), respectively, as shown in Figure 5. Figure 4 presents photographs of the PL emissions. The CIE coordinates of the PL from samples A, B, and C obviously shifted in comparison with that from the reference sample and thus the corresponding emission color changed owing to color mixing with the added emission color that was enabled by the odd SP in the MDM structure. These experimental results indicate that the color mixing of the PL emission can be obtained from a single emitting layer by forming an MDM structure and that the emission color can be tuned by varying the thickness of the emitting middle layer.

## Conclusions

Color-tunable mixed PL emission was achieved by insertion of an emitting layer between two metal layers of finite thickness to form an MDM structure. The PL emission is produced by the combination of two kinds of emission. The first is the organic fluorescent emission; the second is enabled by the odd SP in the MDM structure, and its wavelength is tunable, resulting in a color-tunable mixed emission. This emission process provides a feasible approach to generating two-primary white light.

## Competing interests

The authors declare that they have no competing interests.

## Authors' contributions

NCC and CCL carried out the study of coupled surface plasmon in MDM structure and participated in the sequence alignment and drafted the manuscript. CCC participated in the design of the study and sequence alignment. WTF, JHW, JYL, and SPC fabricated the MDM samples, measured the optical properties, and helped to draft the manuscript. BRH and LLL conceived of the study, participated in its design and coordination, and helped draft the manuscript. All authors read and approved the final manuscript.
